# Child Marriage and Problems Accessing Healthcare in Adulthood: Evidence from India

**DOI:** 10.3390/healthcare10101994

**Published:** 2022-10-11

**Authors:** Biplab Datta, Ajay Pandey, Ashwini Tiwari

**Affiliations:** 1 Institute of Public and Preventive Health, Augusta University, Augusta, GA 30912, USA; 2 Department of Population Health Sciences, Medical College of Georgia, Augusta University, Augusta, GA 30912, USA; 3 Department of Biological Sciences, Augusta University, Augusta, GA 30912, USA

**Keywords:** child marriage, healthcare access, women’s health, India

## Abstract

The association between child marriage and the access to or utilization of maternal and antenatal healthcare has been widely studied. However, little is known about child brides’ access to healthcare for illnesses later in life. Using data on 496,283 married women aged 18 to 49 years from the India National Family and Health Survey 2015–2016, we developed an 11-point composite score (ranging from 0 to 10) outlining the extent of problems accessing healthcare, as follows: (i) no/little problem (score 0 to 2), (ii) some problems (score 3 to 6), and (iii) big problems (score 7 to 10). The differences between child brides and their peers married as adults were assessed by the relative risk ratios obtained from multinomial logistic regressions. The adjusted risk of having “some problems” and “big problems” accessing healthcare relative to “no/little problem” for child brides was found to be 1.22 (95% CI: 1.20–1.25) and 1.26 (95% CI: 1.22–1.29) times that of those married as adults, respectively. These findings highlight the disproportionate barriers to healthcare access faced by women married as children compared to women married as adults and the need for further research to inform policies regarding effective public health interventions to improve healthcare access.

## 1. Introduction

Providing access to and delivering healthcare services to women in developing countries is a global health challenge resulting from the prevailing sociocultural, financial, and legal barriers [1]. While maternal and child healthcare has been the primary focus of women’s healthcare in the developing world, the prevention and control of chronic and noncommunicable diseases (NCDs), especially at different stages of women’s lives, are often neglected [2]. With the growing burden of NCDs in low- and middle-income countries (LMICs) [3], improving women’s health and wellbeing requires a shift from the narrow focus on maternal and child health to a consideration of broader aspects encompassing other disease conditions. Further, identifying the underlying causes of inequities in accessing and utilizing healthcare, particularly in low-resource settings, is critical to inform effective strategies for improving women’s health outcomes. As such, this study examines whether child marriage, a violation of human rights [4], is associated with problems accessing healthcare later in life.

Child marriage, defined as marriage before the age of 18 years, is associated with various forms of adverse health and socioeconomic outcomes, including maternal and child mortality and morbidity, sexually transmitted infections, intimate partner violence, lower educational attainment, and a lack of voice and agency [5,6]. The associations between child marriage and the access to and utilization of maternal healthcare, antenatal care, and institutional delivery have also been widely studied in the existing literature. Compared to women married as adults (i.e., age 18+ years), child brides are less likely to receive antenatal care visits, have a skilled birth attendant present during delivery, give birth in a facility, and attend postnatal care visits [7,8,9,10,11]. However, outside of maternal healthcare utilization, little is known about the relationship between child marriage and the problems faced by women receiving treatment while sick later in life.

**Figure 1:**
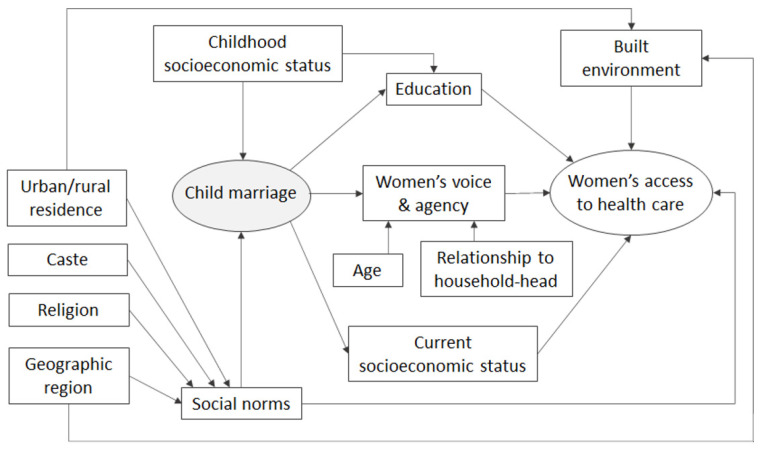
Conceptual framework of the relationship between child marriage and women’s access to healthcare.

Women’s access to non-maternal healthcare services later in life could be impacted by the lingering effects of child marriage. Figure 1 presents the conceptual framework of the relationship between child marriage and women’s access to healthcare, commensurate with the findings in the extant literature on maternal healthcare service utilization and women’s access to HIV treatment and care [12,13]. Education and economic status are directly associated with access to healthcare services [12], and child marriage impacts both educational attainment and women’s current socioeconomic status [5]. Respondents’ childhood socioeconomic status (SES), on the other hand, affects both their education and their likelihood of getting married as child [14,15]. Social norms, including social expectations, gender norms, and religious beliefs, are other important drivers of child marriage [16]. Women’s access to healthcare is also impacted by the prevailing social norms and cultural practices in the community [17,18], which vary across geographic regions and localities. In addition, the built environment (e.g., physical infrastructure) in urban and rural localities across regions can influence women’s healthcare access [19].

The key mechanism through which child marriage may affect women’s access to healthcare is suggested to be a child bride’s limited voice and agency. Child brides have little bargaining power and limited decision-making ability within their marital households, which often remains the case throughout their marriage [5]. This lack of autonomy may also entail a lack of access to and control over household resources. Additionally, seniority within the household, represented by respondents’ age and relationship to the head of the household, may impact women’s participation in decision-making processes [20]. Under this framework, this paper aimed to examine whether women who were married as children face a higher extent of problems accessing healthcare later in life compared to their peers who were married as adults. We examined this issue using nationally representative observational data from India, where child marriage remains a major social problem and a public health concern.

Despite notable progress made in child marriage prevention, India is home to 223 million child brides [21]. Approximately one in four young women in India are married before the age of 18 years [21]. Assessing the relationship between child marriage and problems accessing healthcare, therefore, has important implications for a large share of the female population in India. As in other developing countries, women’s healthcare access in India is primarily studied in the context of maternal healthcare services [22,23,24,25,26]. As such, there is a dearth of evidence on the factors associated with women’s healthcare access beyond maternal healthcare in India. Furthermore, the link between child marriage and problems faced by women accessing general healthcare has not been evaluated in previous studies. This paper contributes to the literature by assessing this critical relationship. Knowledge about whether women married at an early age face greater challenges accessing healthcare later in life has important implications for reducing disparities in healthcare access among women. This study thus has relevance for improving the health and wellbeing of child brides in India.

## 2. Materials and Methods

### 2.1. Data

We used data on 496,283 married women aged 18 to 49 years from the 2015–2016 wave of the India National Family and Health Survey (NFHS-4). The NFHS-4 is a nationally representative survey that provides various sociodemographic and health-related data from reproductive-aged women, covering the 36 states and union territories and 640 districts of India [27]. Participation in the NFHS-4 was voluntary, and informed consent was obtained prior to each interview. The survey protocols of the NFHS-4 were reviewed and approved by the Institutional Review Boards of the International Institute for Population Sciences and the ICF [27].

### 2.2. Measures

In the NFHS-4, respondents were asked about factors that they considered to be no problem, a small problem, or a big problem in relation to accessing medical advice or treatment while being sick. The factors included: (i) obtaining permission, (ii) acquiring money, (iii) commuting (distance to the health facility and need for transportation), (iv) having a personal attendant to go with, and (v) concerns about the facility (unavailability of female provider and essential drugs). Notably, the two factors included under “commuting” and the two factors included under “concerns about the facility” were consolidated into single factors due to their high correlation.

The response options for each factor were coded as follows: 0 for “no problem”, 1 for “small problem”, and 2 for “big problem”. To accommodate the multi-faceted nature of the problems related to accessing healthcare, we aggregated all coded responses to develop an 11-point composite score (ranging from 0 to 10) of the degree of problems faced when accessing healthcare. Based on the percentile values of the score, we defined three categories of difficulty accessing healthcare, as follows: (i) no/little problem (≤25th percentile: score 0 to 2); (ii) some problems (>25th and ≤75th percentile: score 3 to 6); and (iii) big problems (>75th percentile: score 7 to 10).

We examined the validity of the constructed score by assessing the likelihood of receiving treatment for certain chronic conditions across the three categories of healthcare access problems. The NFHS-4 asked the respondents if they had (at the time of the survey) the following disease conditions—diabetes, asthma, thyroid disorder, any heart disease, or cancer. If a respondent indicated that they had a disease condition, she was further asked if treatment had been sought for that condition. Using this information, we estimated the share of women with a disease condition not seeking treatment for the problem. The composite score was regarded as consistent if the share was lower for the “no/little problem” group and higher for the “big problems” group.

To further assess the degree of the problems faced when accessing healthcare, we considered another outcome, measured by the number of “big problems” for individual factors, as follows: (i) 1 big problem, (ii) 2 big problems, and (iii) 3+ big problems accessing healthcare.

Our key explanatory variable was a binary variable indicating whether the respondent was married before the age of 18. Other covariates in the multivariable model included: age—18 to 29 (reference group), 30 to 44, or 45 to 49; residence—rural (reference group) or urban; household wealth—poor (reference group) or non-poor; religion—Hindu (reference group), Muslim, Christian, or other; caste—non socially backward class (reference group), scheduled caste, scheduled tribe, or other backward class; relationship to household head—head (reference group), wife, daughter, daughter-in-law, or other; and geographic region fixed effects.

The socioeconomic status (SES) was captured by a categorical variable that entailed the women’s educational attainment and household wealth, as follows: (i) poor—lower education; (ii) poor—higher education; (iii) non-poor—lower education; and (iv) non-poor—higher education (reference group). Poor was defined as the bottom two quintiles of the household wealth index, and non-poor was defined as the top three quintiles of the household wealth index. Lower education was defined as primary or no education, and higher education was defined as a secondary or higher level of education.

### 2.3. Statistical Analysis

We first compared the proportion of women facing different degrees of problems accessing healthcare for the five individual factors across the two groups—married as adults vs. married as children. Next, we estimated multinomial logistic regression models to obtain relative risk ratios in favor of experiencing “small problems” and “big problems”, relative to the base outcome of “no problem”, for each of the five factors, namely (i) permission, (ii) money, (iii) commute, (iv) personal attendant, and (v) facility. We obtained both adjusted and unadjusted ratios by estimating models with and without the model covariates.

We then compared the proportion of women in the two groups facing “no/little problem”, “some problems”, and “big problems” accessing healthcare based on the 11-point composite score. We also compared the groups across various sociodemographic subgroups, based on age, urban/rural residence, religion, caste, relationship to household head, and geographic region. We performed weighted Wald tests to examine statistically significant differences between the two groups.

To assess the relationship between our composite score of problems faced when accessing healthcare and child marriage, we first estimated a linear model. In this model, the outcome was the 11-point continuous composite score, and the key explanatory variable was the child marriage indicator. A positive and statistically significant coefficient estimate of the child marriage indicator in this specification was suggestive of greater problems accessing healthcare for the child brides compared to their counterparts who were married as adults.

Next, we estimated another multinomial logistic regression model to obtain the relative risk ratios in favor of facing “some problems” and “big problems”, relative to the base outcome of “no/little problem”, accessing healthcare. The exponentiated value of the coefficient estimate of the child marriage indicator for the respective outcomes reflected how the risks differed between women married as children and as adults. For both the linear model and the multinomial logistic model, we also obtained adjusted coefficient estimates by accounting for several sociodemographic covariates (age, residence, religion, caste, relationship to household head, and geographic region). However, we did not include these correlates in the model to assess their relationship to problems accessing healthcare; rather, we intended to estimate the adjusted relative risks for the child marriage indicator after controlling for the relevant sociodemographic characteristics of the respondents, as outlined in the conceptual framework.

Next, in addition to the sociodemographic correlates, we controlled for women’s SES conditions in the model. As outlined in the conceptual framework, the current SES conditions were potential channels of the relationship between child marriage and women’s access to healthcare. As such, the estimated coefficients for the child marriage indicator in both the linear and multinomial logistic models were expected to decrease and/or become statistically insignificant after controlling for the SES conditions.

Since we were interested in healthcare access beyond maternal and child healthcare, we further estimated a model for the subgroup of women who were not currently pregnant or lactating and had not given birth in the five years preceding the survey. Additionally, to check the robustness of our results, we estimated models for the urban and rural subgroups. As an alternative strategy to assess healthcare access beyond maternal and child care by fertility differentials across age groups, we also estimated the models for the following age-based subgroups: (i) 18 to 34 years, (ii) 35 to 44 years, and (iii) 45 to 49 years.

Lastly, to assess the degree of the problems faced by those who experienced “big problems”, we estimated a two-part hurdle model. The first part examined whether the respondent faced any “big problem” accessing healthcare, and the second part examined the extent (1, 2, or 3+) of the “big problems” among those who had indicated at least one. We estimated a binomial logistic regression model for the first part and a truncated Poisson regression model for the second part. Statistical analyses were conducted using Stata 17.0 software (College Station, TX, USA), and the level of significance was set at 5% (i.e., α = 0.05). All estimates were obtained using complex survey weights entailing the two-stage stratified sampling framework of the NFHS-4, applying the “svy” command in Stata.

## 3. Results

Approximately 44% of the women in the study sample were married before the age of 18 years. Table 1 presents the background characteristics of the study participants grouped by marriage age. Women residing in rural areas, having lower educational attainment, and from poor households were more likely to have been married as children. The incidence of child marriage was also higher among women who identified themselves as belonging to a scheduled caste or tribe and who lived in the eastern part of India.

Child brides reported that they faced a “big problem” at a greater frequency and “no problem” at a lower frequency compared to women married as adults for all five factors affecting healthcare access (Table 2). Approximately 17% of the women married as adults reported “obtaining permission” as a “big problem”. This estimate was two percentage points higher among child brides. Conversely, while nearly 50% of the women married as adults mentioned that “acquiring money” was not a problem, this proportion was ten percentage points lower among women married as children. Relative to the base outcome of “no problem”, the relative risk ratios in favor of facing a “small problem” or a “big problem” for individual factors (i.e., permission, money, commute, personal attendant, and facility) are presented in Table 3. For all five individual factors, child brides had a higher adjusted relative risk of facing a “big problem” than their counterparts who were married as adults.

**Figure 2:**
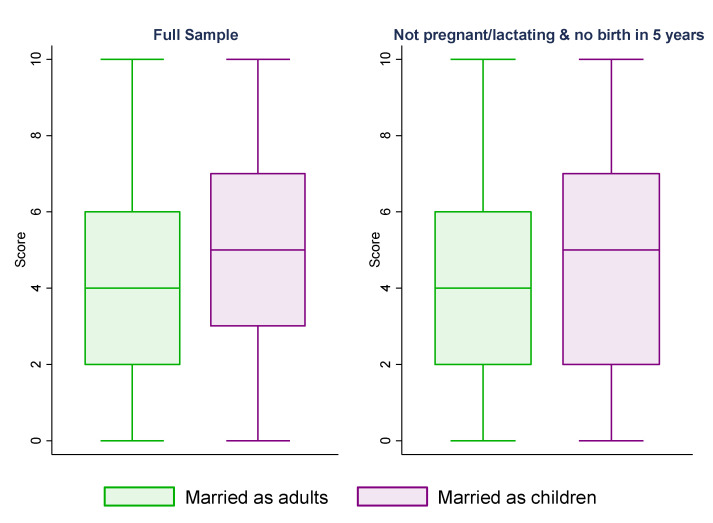
Relationship between marriage age and 11-point score.

Figure 2 presents box plots showing the variations in the composite score between the “married as adults” and “married as children” groups. While the 25th, 50th, and 75th percentiles of the score for women who were married as adults were 2, 4, and 6, respectively, they were 3, 5, and 7 for women who were married as children. This suggested a positive association between child marriage and the degree of difficulties faced when accessing healthcare.

**Figure 3:**
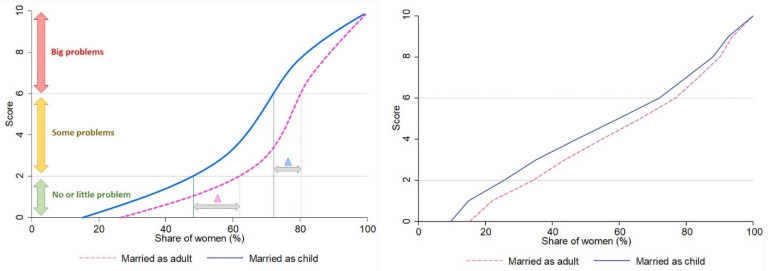
Distribution of healthcare access problem severity (based on composite score) by marriage age. The figure on the left illustrates the analytical framework, and the figure on the right presents the actual distribution in the sample.

Figure 3 illustrates the cumulative distribution of the composite score between women married as children and as adults. It shows significant differences in the proportion of women facing “no/little problem” and “big problems”. While only 25.6% of the child brides reported “no/little problem” accessing healthcare, the share was higher (34.2%) among their peers who were married as adults. Conversely, 23.3% of the women who were married as adults faced “big problems” accessing healthcare, whereas that share among child brides was greater (28.1%).

**Figure 4:**
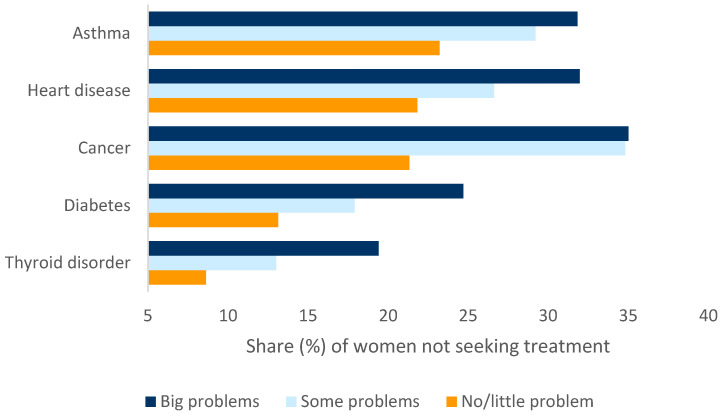
Validity check for the distribution of healthcare access problem severity (based on composite score). Sample for validity check contained women who reported certain disease conditions. Among them, the share of women who were (at the time of the survey) not seeking treatment for a problem is plotted on the horizontal axis against the respective disease condition and the severity of the healthcare access problems faced.

Figure 4 presents the validation results of the composite score. For each of the five chronic conditions, women who faced “big problems” accessing healthcare (i.e., composite score ≥ 6) were less likely to seek treatment compared to women who faced “no/little problem” (i.e., composite score ≤ 2). For example, among women with a thyroid disorder, the rate of not seeking treatment was around 9% for those who experienced “no/little problem”, whereas it was around 19% for those who experienced “big problems”. Thus, the validation exercise suggested that the higher the composite score, the lower the likelihood of seeking treatment.

The share of women facing “no/little problem”, “some problems”, and “big problems” based on the composite score according to marriage age and sociodemographic characteristics is presented in Table 4. The difference in the share of women facing “big problems” among child brides and women married as adults gradually declined with age. The difference was six percentage points in the youngest age group (18 to 29 years), while it was only two percentage points in the oldest age group (45 to 49). The difference was higher in urban areas than in rural areas (4.7 vs. 2.5 percentage points). Compared to women who were household heads or the wife of the household head, the difference was also higher among women whose relationship with the household head was daughter or daughter-in-law. The higher share of child brides facing “big problems” was also evident across various religious groups, castes, and geographic regions.

The linear relationship between the composite score and the child marriage indicator and the relative risk ratios (RRR) and adjusted relative risk ratios (ARRR) in favor of facing “some problems” or “big problems” relative to the base outcome of “no/little problem” are presented in Table 5. The estimates of the linear model indicated a positive relationship between child marriage and the composite score. The results of the multinomial logistic regression suggested that the relative risk of experiencing “big problems” accessing healthcare relative to “no/little problem” for child brides was 1.62 (95% CI: 1.57–1.66) times that of those who were married as adults. The adjusted relative risk was, however, slightly lower—1.26 (95% CI: 1.22–1.29) times that of those who were married as adults. The results were very similar for the subgroup of those who were not pregnant or lactating and who had not given birth in the past 5 years. When the SES conditions were accounted for, the relationship estimated by the linear model became 0.057, which was much smaller than the figure of 0.247 obtained without the SES conditions. Similarly, the ARRRs for “some problems” and “big problems” also decreased when SES conditions were controlled for in the model. These results suggested that SES conditions were potential channels of the relationship between child marriage and women’s access to healthcare.

Table 6 presents the results according to the urban and rural subgroups. The ARRRs in favor of experiencing “big problems” for the child marriage indicator were 1.50 (95% CI: 1.41–1.60) and 1.16 (95% CI: 1.12–1.19), respectively, in the urban and rural subsamples. The results by age cohort are presented in Table 7. The adjusted relative risk in favor of facing “big problems” among child brides was 1.09 to 1.34 times that of their counterparts married as adults across the age groups. The results were thus robust across various subgroups and specifications. The ARRRs for “big problems” in the rural subgroup, however, were not statistically significant when SES conditions were controlled for.

The results according to different age groups related to different fertility rates were presented in Table 7. Relative to “no/little problem”, the adjusted risk of facing “big problems” for child brides was 1.32 (95% CI: 1.27–1.38) and 1.27 (95% CI: 1.22–1.32), respectively, for women aged 18 to 28 years and 30 to 44 years. The ARRR, however, was not statistically significant for women aged 45 to 49 years. When the SES conditions were controlled for, child brides aged 45 to 49 years were found to be less likely to face “big problems” compared to their similarly aged peers who were married as adults.

Lastly, Table 8 presents the results of the hurdle model. The adjusted odds ratio in favor of facing at least one “big problem” among child brides was 1.16 (95% CI: 1.13–1.18) times that of women who were married as adults. The incidence rate ratios in favor of experiencing “big problems” from the truncated Poisson model were statistically significant and greater than one, indicating that child brides faced “big problems” at a greater rate compared to their counterparts married as adults.

## 4. Discussion

This paper investigated the relationship between child marriage (i.e., before the age of 18 years) and difficulties accessing healthcare outside the scope of maternal and child healthcare services while sick later in life. Our findings suggested that child brides were more likely to have problems accessing healthcare compared to their peers married as adults. As such, child marriage was associated with a greater degree of barriers to accessing healthcare. This has critical population health implications, as several recent studies indicate a higher risk of chronic conditions among child brides during adulthood [28,29,30,31,32]. Having a higher risk of chronic conditions, accompanied by a higher degree of difficulties accessing non-maternal healthcare, may thus heighten the risk of adverse health outcomes for child brides as adults.

As noted in the conceptual framework, one potential contributor to problems accessing non-maternal healthcare services may be social norms, inclusive of gender inequalities stemming from several social demographic correlates, such as religion, caste, urban/rural residence, and geographic region [33]. Disparities in healthcare access in India are a gendered phenomenon, with women often facing increased barriers to accessing healthcare for themselves [34]. A recent study at a public tertiary referral hospital in India showed that the male-to-female outpatient visit ratio was 1.69, despite the population of India having an overall sex ratio of 1.09 [35]. However, after controlling for the available determinants contributing to gender inequalities, we continued to observe a persistent relationship between child marriage and difficulties accessing healthcare.

While not directly measurable in this study, it is plausible that gender inequalities continue propagate into adulthood, particularly among child brides. For example, one proposed mechanism, women’s empowerment, as illustrated in the conceptual framework, has been linked to the uptake of contraceptive use and the greater utilization of maternal healthcare [36,37,38,39]. Studies have shown that child brides have decreased autonomy and bargaining power within their household, as well as limited or no access to household funds [5,40,41]. Child brides, compared to their peers married as adults, experience more controlling behaviors from their husband and their husband’s family [42] and bear a greater risk of experiencing IPV [43,44], which could further exacerbate obstacles accessing health services, as seen in studies related to maternal and child healthcare [45].

An interesting finding of our analysis was the lower likelihood of experiencing “big problems” accessing healthcare for the child brides at age 45 to 49 years, compared to their similarly aged peers who were married as adults, when the SES conditions were accounted for. This may be attributable to the seniority of women within the household impacting their decision-making ability. Women’s autonomy regarding decision making is an important determinant of healthcare access. A recent meta-analysis reported that women’s age, educational attainment, place of residence, and economic conditions were critical determinants of their decision-making autonomy in relation to the access to and utilization of maternal health services [46]. Evidence in the extant literature, particularly in low-resource settings, further suggests that the opinion of the husband or mother-in-law is an important factor in the decision to access maternal healthcare [47,48]. A recent study found that women in Myanmar with high levels of empowerment, as measured by decision-making power within the household and disagreement with the justification of wife beating, were less likely to face barriers accessing healthcare compared to women with lower levels of empowerment [49]. 

Intertwined is educational attainment, where higher education acts as a protective factor in decision-making and healthcare-seeking behaviors [12,49,50]. A scoping review suggested that limited access to healthcare information is another major impediment to accessing healthcare services for women of reproductive age in LMICs [51]. Child brides are more likely to have lower literacy rates, higher primary and secondary school dropout rates, and overall lower levels of education compared to women married as adults [52,53]. In addition to the lack of knowledge and awareness, transportation-related barriers and the unequal provision of healthcare facilities at different socioeconomic levels were identified as major obstacles to accessing maternal healthcare in many LMICs [54]. Child marriage, by limiting women’s labor force participation and ability to engage in activities outside the household, [5] may impose added barriers to commuting to the health facilities.

A recent study that examined the individual and community-level factors affecting women’s access to healthcare services in Benin, a West African country, found that socioeconomic conditions such as economic status and the partner’s educational attainment were significant predictors of problems faced by women accessing healthcare [55]. At the community level, the study found that the literacy level in the community was significantly associated with problems related to healthcare access [55]. Other similar studies using data from Ghana, Tanzania, and Ethiopia outlined low socioeconomic status as one of the key barriers to women’s access to healthcare [56,57,58]. A study on 24 countries in sub-Saharan Africa reported similar findings [59]. In addition to low socioeconomic status, a lack of health insurance coverage and limited exposure to mass media was also found to be associated with problems accessing healthcare for women in LMICs [60,61]. Further, these barriers were found to be disproportionately concentrated among women from low-income households [61]. Child marriage adversely impacts women’s economic opportunities [5], resulting in lower economic status for women who were married as children. Child brides also experience limited access to mass media [62,63]. Taken together, these studies illustrate the complexity of social, family, and gender dynamics in potentially affecting access to healthcare services for women who were married as children.

Unlike the various initiatives taken to improve access to maternal and child healthcare [64], little action has been taken to improve access to general healthcare for women. Furthermore, studies on the inequities in and barriers to women’s healthcare utilization in India have primarily focused on antenatal care services [65,66]. Our analyses thus have important implications for improving healthcare utilization among women in India. Our findings suggested that in addition to the gender disparity in accessing healthcare, women in India face disparities in accessing healthcare services based on the age at which they were married. These findings motivate the need for additional research in this area to inform policies that aim to reduce the overall disparity in healthcare access in the Indian population.

Child marriage is a social problem that has deep roots in social norms and cultural practices [67]. Eradicating child marriage, therefore, requires culturally sensitive community-level initiatives supported by other programmatic interventions and legal instruments [68]. Sociocultural norms and gender stereotypes are factors that also affect women’s healthcare access [69]. Further, a lack of culturally appropriate intervention strategies and cultural dissonance in program design can impede improvement in healthcare access among marginalized women in LMICs [70,71]. As such, culturally acceptable community-based interventions to empower women and promoting supportive structures within families may be helpful in improving a child bride’s access to healthcare [72]. Social policies aimed at promoting social networks and engendering social capital within the community may help as well [73,74]. In addition, national-level policies to promote gender equality and structural interventions to create economic opportunities for women will facilitate child brides’ healthcare access in LMICs [75].

This work should be considered in the context of several limitations. Given the cross-sectional nature of the data, we were unable to identify a causal relationship between child marriage and problems accessing healthcare. The questions asked in the survey about problems accessing healthcare were generic in nature and did not provide a specific reference period. Data on the gravity of sickness were also not available. Further, we could not empirically assess the role of voice and agency through which child marriage may impact healthcare access. Future research, with appropriate data, may examine these issues to provide a nuanced understanding of this relationship. The current study was focused on child marriage among girls and did not explore the potential consequences of child marriage among boys. Another direction for future research could be investigating the impact of child marriage among boys on their and their spouses’ health and healthcare access later in life.

## 5. Conclusions

Our analyses in this study provided a novel contribution to the literature on women’s healthcare access in LMICs by exploring a less visited domain of general healthcare, beyond maternal and child care. Our results suggested that women married as children face a greater degree of difficulties accessing healthcare compared to women who were married as adults. These findings were robust across urban and rural areas as well as across different age groups. As such, further studies are warranted to design strategic interventions to ensure adequate healthcare access later in life for women who were married as children.

## Figures and Tables

**Table 1:** Background characteristics of the study participants grouped by marriage age.

	Frequency			Share ^1^		
	All	Married as Adults	Married as Children	All	Married as Adults	Married as Children
Child marriage	206,196	0	206,196	43.92	0.00	100.00
**Covariates**						
Age						
18–29	190,750	116,711	74,039	38.93	41.29	35.92
30–44	242,565	137,270	105,295	48.33	46.32	50.91
45–49	62,968	36,106	26,862	12.73	12.39	13.17
Residence						
Rural	357,509	198,035	159,474	66.48	61.20	73.23
Urban	138,774	92,052	46,722	33.52	38.80	26.77
Religion						
Hindu	376,867	213,213	163,654	81.45	80.61	82.52
Muslim	63,347	36,276	27,071	13.13	12.59	13.83
Christian	32,682	23,532	9150	2.23	2.80	1.51
Other	23,387	17,066	6321	3.19	4.00	2.15
Caste						
None	123,203	80,240	42,963	26.98	29.61	23.63
Scheduled caste	88,997	47,539	41,458	20.23	18.55	22.38
Scheduled tribe	86,268	51,463	34,805	9.11	8.28	10.16
Other backward class	197,815	110,845	86,970	43.68	43.56	43.83
Relationship to household head						
Head	19,776	10,094	9682	4.00	3.41	4.75
Wife	311,428	167,647	143,781	62.76	57.42	69.57
Daughter	26,019	17,957	8062	5.54	6.67	4.10
Daughter-in-law	120,745	82,920	37,825	23.88	28.38	18.13
Other	18,315	11,469	6846	3.83	4.12	3.45
Region						
North	99,605	65,302	34,303	13.48	15.22	11.25
Central	129,879	67,371	62,508	22.65	21.15	24.56
East	93,224	47,165	46,059	22.93	19.72	27.04
Northeast	61,862	41,458	20,404	3.07	3.32	2.74
West	41,108	24,940	16,168	14.42	15.47	13.09
South	70,605	43,851	26,754	23.45	25.12	21.32
**Socioeconomic status**						
Education						
Low	244,581	111,635	132,946	47.47	35.83	62.34
High	251,702	178,452	73,250	52.53	64.17	37.65
Household wealth						
Poor	204,794	100,593	104,201	37.72	30.81	46.55
Non-poor	291,489	189,494	101,995	62.28	69.19	53.45
Total	496,283	290,087	206,196	100.00	100.00	100.00

^1^ Estimates were obtained using complex survey weights.

**Table 2:** Share of women reporting different degrees of problems for each factor according to marriage age.

	Share of Women Facing Problems Accessing Healthcare (%) ^1^					
	No Problem		Small Problem		Big Problem	
	Married as Adults	Married as Children	Married as Adults	Married as Children	Married as Adults	Married as Children
Permission	61.10	58.73	22.18	22.45	16.72	18.82
	(60.64, 61.57)	(58.24, 59.22)	(21.81, 22.55)	(22.09, 22.82)	(16.38, 17.06)	(18.44, 19.19)
Money	48.74	39.97	28.73	31.09	22.53	28.94
	(48.29, 49.20)	(39.51, 40.43)	(28.36, 29.10)	(30.70, 31.49)	(22.16, 22.90)	(28.50, 29.38)
Commute	35.03	26.37	33.05	34.23	31.92	39.39
	(34.57, 35.49)	(25.94, 26.81)	(32.67, 33.43)	(33.82, 34.64)	(31.51, 32.33)	(38.90, 39.88)
Attendant	25.62	20.25	22.50	22.50	51.88	57.26
	(25.16, 26.07)	(19.83, 20.67)	(22.15, 22.85)	(22.11, 22.89)	(51.41, 52.36)	(56.74, 57.77)
Facility	50.98	45.03	31.15	33.78	17.87	21.19
	(50.52, 51.44)	(44.56, 45.50)	(30.77, 31.53)	(33.39, 34.18)	(17.55, 18.19)	(20.82, 21.55)

^1^ Number of women = 496,283. Number of women married as adults = 290,087. Number of women married as children = 206,196. For women married as adults, columns (1), (3), and (5) add up to 100%. For women married as children, columns (2), (4), and (6) add up to 100%. Estimates were obtained using complex survey weights; 95% confidence intervals are in parenthesis. The differences between the “married as adults” and “married as children” groups were statistically significant (*p* < 0.01) for all five factors for “no problem” and “big problems”, and for the money, commute, and facility factors for “small problem”.

**Table 3:** Estimates of adjusted relative risk ratios for facing specific problems accessing healthcare.

	Adjusted without SES ^1^			Adjusted for SES ^2^		
	Base Outcome:	Outcome I:	Outcome II:	Base Outcome:	Outcome I:	Outcome II:
	No Problem	Small Problem	Big Problem	No Problem	Small Problem	Big Problem
**A. Permission**						
Child marriage	Ref.	1.030 *	1.081 ***	Ref.	0.974 **	0.973 **
		(1.006, 1.055)	(1.053, 1.109)		(0.951, 0.997)	(0.948, 0.998)
**B. Money**						
Child marriage	Ref.	1.186 ***	1.268 ***	Ref.	1.081 ***	1.081 ***
		(1.160, 1.213)	(1.237, 1.300)		(1.057, 1.106)	(1.054, 1.109)
**C. Commute**						
Child marriage	Ref.	1.184 ***	1.277 ***	Ref.	1.066 ***	1.086 ***
		(1.155, 1.214)	(1.245, 1.311)		(1.040, 1.092)	(1.058, 1.115)
**D. Attendant**						
Child marriage	Ref.	1.142 ***	1.151 ***	Ref.	1.068 ***	1.053 ***
		(1.107, 1.178)	(1.120, 1.182)		(1.035, 1.101)	(1.026, 1.082)
**E. Facility**						
Child marriage	Ref.	1.115 ***	1.131 ***	Ref.	1.037 ***	1.007
		(1.092, 1.138)	(1.103, 1.160)		(1.016, 1.059)	(0.982, 1.033)

^1^ Estimates were obtained using complex survey weights; 95% confidence intervals are in parenthesis; *** *p* < 0.001, ** *p* < 0.01, * *p* < 0.05. The multivariable specifications accounted for age group (18 to 29, 30 to 44, or 45 to 49); residence (urban or rural); religion (Hindu, Muslim, Christian, or other); caste (none, scheduled caste, scheduled tribe, or other backward class); relationship to household head (head, wife, daughter, daughter-in-law, or other); and geographic region fixed effects (north, central, east, northeast, west, or south). ^2^ In the SES specification, in addition to the sociodemographic correlates, the following SES conditions were controlled for: poor and lower education, poor and higher education, non-poor and lower education, and non-poor and higher education.

**Table 4:** Share of women facing problems accessing healthcare according to degree of problems, marriage age, and sociodemographic characteristics.

	Share of Women Facing Problems Accessing Healthcare (%) ^1^					
	No/Little Problem		Some Problems		Big Problems	
	Married as Adults	Married as Children	Married as Adults	Married as Children	Married as Adults	Married as Children
**All**	34.22	25.56	42.52	46.38	23.26	28.06
	(33.75, 34.70)	(25.13, 25.99)	(42.10, 42.94)	(45.93, 46.82)	(22.87, 23.64)	(27.62, 28.51)
**Age**						
18 to 29	31.81	22.66	43.44	46.60	24.76	30.74
	(31.25, 32.36)	(22.11, 23.22)	(42.91, 43.96)	(45.97, 47.22)	(24.28, 25.23)	(30.11, 31.37)
30 to 44	36.19	26.55	41.83	46.46	21.98	26.98
	(35.62, 36.77)	(26.04, 27.07)	(41.32, 42.34)	(45.94, 46.99)	(21.54, 22.42)	(26.48, 27.49)
45 to 49	34.92	29.61	42.06	45.45	23.02	24.95
	(34.03, 35.82)	(28.72, 30.49)	(41.19, 42.92)	(44.53, 46.37)	(22.28, 23.76)	(24.15, 25.74)
**Residence**						
Rural	25.82	21.27	45.44	47.46	28.74	31.27
	(25.40, 26.24)	(20.84, 21.70)	(45.01, 45.86)	(46.98, 47.93)	(28.31, 29.18)	(30.77, 31.78)
Urban	47.48	37.29	37.92	43.43	14.6	19.28
	(46.48, 48.49)	(36.19, 38.39)	(37.06, 38.78)	(42.40, 44.46)	(13.94, 15.26)	(18.34, 20.23)
**Religion**						
Hindu	33.51	25.28	42.80	46.51	23.69	28.21
	(33.00, 34.01)	(24.82, 25.74)	(42.35, 43.26)	(46.04, 46.99)	(23.27, 24.11)	(27.73, 28.68)
Muslim	33.99	26.53	42.24	45.87	23.77	27.6
	(32.80, 35.18)	(25.39, 27.68)	(41.23, 43.26)	(44.62, 47.11)	(22.86, 24.68)	(26.43, 28.76)
Christian	48.29	30.08	33.81	43.23	17.9	26.69
	(46.13, 50.46)	(26.66, 33.50)	(32.02, 35.59)	(40.24, 46.22)	(16.27, 19.53)	(23.60, 29.78)
Other	39.56	26.69	43.79	46.67	16.65	26.64
	(37.60, 41.52)	(23.82, 29.56)	(41.94, 45.64)	(44.21, 49.14)	(15.34, 17.96)	(24.07, 29.20)
**Caste**						
None	43.04	28.67	39.42	47.24	17.54	24.09
	(42.18, 43.90)	(27.76, 29.58)	(38.70, 40.14)	(46.31, 48.17)	(16.95, 18.13)	(23.22, 24.95)
Scheduled caste	27.26	22.99	45.90	47.35	27.20	29.66
	(26.43, 28.09)	(22.23, 23.75)	(44.78, 47.02)	(46.45, 48.26)	(26.37, 28.02)	(28.80, 30.52)
Scheduled tribe	19.37	17.33	42.70	46.42	34.73	36.25
	(18.42, 20.31)	(16.29, 18.37)	(42.16, 43.24)	(45.24, 47.59)	(33.59, 35.88)	(35.08, 37.42)
Other backward class	34.02	27.10	39.42	45.41	23.28	27.50
	(33.42, 34.62)	(26.53, 27.67)	(38.70, 40.14)	(44.86, 45.95)	(22.80, 23.76)	(26.93, 28.06)
**Relationship to household head**						
Head	27.22	21.55	42.73	45.23	30.05	33.22
	(25.82, 28.62)	(20.35, 22.76)	(41.27, 44.19)	(43.81, 46.64)	(28.65, 31.45)	(31.84, 34.60)
Wife	33.17	25.90	43.15	46.48	23.69	27.62
	(32.60, 33.74)	(25.42, 26.38)	(42.63, 43.66)	(46.00, 46.97)	(23.23, 24.14)	(27.13, 28.11)
Daughter	36.79	24.24	40.25	46.33	22.96	29.43
	(35.63, 37.95)	(22.92, 25.56)	(39.10, 41.40)	(44.82, 47.84)	(22.00, 23.92)	(28.05, 30.82)
Daughter-in-law	36.48	25.58	41.84	46.15	21.68	28.27
	(35.85, 37.10)	(24.84, 26.31)	(41.24, 42.44)	(45.32, 46.98)	(21.19, 22.17)	(27.52, 29.03)
Other	35.06	25.66	41.98	47.11	22.96	27.23
	(33.64, 36.49)	(24.16, 27.16)	(40.61, 43.35)	(45.33, 48.89)	(21.74, 24.17)	(25.67, 28.79)
**Region**						
North	38.01	28.14	43.60	49.63	18.39	22.23
	(37.03, 38.99)	(27.01, 29.28)	(42.52, 44.68)	(48.23, 51.03)	(17.66, 19.13)	(21.24, 23.21)
Central	32.20	26.14	45.09	47.24	22.71	26.62
	(31.46, 32.94)	(25.46, 26.82)	(44.44, 45.74)	(46.61, 47.87)	(22.11, 23.31)	(26.00, 27.23)
East	18.81	14.20	46.72	49.44	34.47	36.35
	(17.98, 19.65)	(13.54, 14.87)	(45.86, 47.59)	(48.49, 50.40)	(33.57, 35.36)	(35.37, 37.33)
Northeast	26.63	19.87	46.51	48.19	26.86	31.94
	(25.44, 27.82)	(18.59, 21.16)	(45.28, 47.73)	(46.70, 49.67)	(25.55, 28.17)	(30.24, 33.63)
West	36.17	28.98	44.38	47.99	19.45	23.03
	(34.74, 37.61)	(27.49, 30.46)	(43.05, 45.71)	(46.56, 49.42)	(18.30, 20.59)	(21.69, 24.38)
South	45.53	36.55	34.74	38.56	19.73	24.89
	(44.34, 46.73)	(35.38, 37.73)	(33.83, 35.64)	(37.62, 39.49)	(18.85, 20.61)	(23.73, 26.05)

^1^ Number of women = 496,283. Number of women married as adults = 290,087. Number of women married as children = 206,196. For women married as adults, columns (1), (3), and (5) add up to 100%. For women married as children, columns (2), (4), and (6) add up to 100%. Estimates were obtained using complex survey weights; 95% confidence intervals are in parenthesis. The differences in the share of women facing problems accessing healthcare between the two groups (i.e., married as adults and married as children) were statistically significant (*p* < 0.01) across all sociodemographic characteristics, except for the difference in “some problems” for the “other religion” category.

**Table 5:** Estimates of linear regression and relative risk ratios for facing problems accessing healthcare categorized by score.

	Linear Model	Multinomial Logistic Model		
		Base Outcome:	Outcome I:	Outcome II:
		No/Little Problem	Some Problems	Big Problems
**A. Unadjusted** ^1^				
I. Full sample (N = 496,283)				
Child marriage	0.558 ***	Ref.	1.460 ***	1.616 ***
	(0.527, 0.588)		(1.427, 1.495)	(1.573, 1.660)
II. Not pregnant/lactating and had not given birth in the past 5 years (N = 283,360)				
Child marriage	0.523 ***	Ref.	1.428 ***	1.557 ***
	(0.485, 0.561)		(1.388, 1.470)	(1.505, 1.610)
**B. Adjusted** ^2^				
I. Full sample (N = 496,283)				
Child marriage	0.247 ***	Ref.	1.224 ***	1.256 ***
	(0.219, 0.276)		(1.196, 1.254)	(1.222, 1.291)
II. Not pregnant/lactating and had not given birth in the past 5 years (N = 283,360)				
Child marriage	0.228 ***	Ref.	1.197 ***	1.225 ***
	(0.192, 0.264)		(1.162, 1.233)	(1.183, 1.268)
**B. Adjusted for SES** ^3^				
I. Full sample (N = 496,283)				
Child marriage	0.057 ***	Ref.	1.090 ***	1.053 ***
	(0.030, 0.085)		(1.064, 1.115)	(1.024, 1.082)
II. Not pregnant/lactating and had not given birth in the past 5 years (N = 283,360)				
Child marriage	0.048 ***	Ref.	1.068 ***	1.038 **
	(0.013, 0.083)		(1.037, 1.100)	(1.003, 1.075)

^1^ Estimates were obtained using complex survey weights; 95% confidence intervals are in parenthesis; *** *p* < 0.001, ** *p* < 0.01 ^2^ The multivariable specifications accounted for age group (18 to 29, 30 to 44, or 45 to 49); residence (urban or rural); religion (Hindu, Muslim, Christian, or other); caste (none, scheduled caste, scheduled tribe, or other backward class); relationship to household head (head, wife, daughter, daughter-in-law, or other); and geographic region fixed effects (north, central, east, northeast, west, or south). ^3^ In the SES specification, in addition to the sociodemographic correlates, the following SES conditions were controlled for: poor and lower education, poor and higher education, non-poor and lower education, and non-poor and higher education.

**Table 6:** Estimates of linear regression and relative risk ratios for facing problems accessing healthcare categorized by score—according to place of residence.

	Linear Model	Multinomial Logistic Model		
		Base Outcome:	Outcome I:	Outcome II:
		No/little Problem	Some Problems	Big Problems
**A. Urban—unadjusted** ^1^				
I. Full sample (N = 138,774)				
Child marriage	0.614 ***	Ref.	1.458 ***	1.682 ***
	(0.551, 0.676)		(1.396, 1.523)	(1.585, 1.785)
II. Not pregnant/lactating and had not given birth in the past 5 years (N = 84,805)				
Child marriage	0.584 ***	Ref.	1.402 ***	1.650 ***
	(0.509, 0.659)		(1.330, 1.478)	(1.535, 1.773)
**B. Rural—unadjusted**				
I. Full sample (N = 357,509)				
Child marriage	0.295 ***	Ref.	1.268 ***	1.321 ***
	(0.265, 0.326)		(1.235, 1.301)	(1.283, 1.359)
II. Not pregnant/lactating and had not given birth in the past 5 years (N = 198,555)				
Child marriage	0.241 ***	Ref.	1.236 ***	1.252 ***
	(0.203, 0.280)		(1.197, 1.277)	(1.207, 1.298)
**C. Urban—adjusted** ^2^				
I. Full sample (N = 138,774)				
Child marriage	0.461 ***	Ref.	1.322 ***	1.502 ***
	(0.398, 0.524)		(1.265, 1.382)	(1.413, 1.597)
II. Not pregnant/lactating and had not given birth in the past 5 years (N = 84,805)				
Child marriage	0.434 ***	Ref.	1.264 ***	1.475 ***
	(0.358, 0.510)		(1.199, 1.333)	(1.370, 1.588)
**D. Rural—adjusted**				
I. Full sample (N = 357,509)				
Child marriage	0.148 ***	Ref.	1.153 ***	1.156 ***
	(0.118, 0.178)		(1.123, 1.184)	(1.122, 1.190)
II. Not pregnant/lactating and had not given birth in the past 5 years (N = 198,555)				
Child marriage	0.114 ***	Ref.	1.134 ***	1.115 ***
	(0.076, 0.152)		(1.096, 1.172)	(1.075, 1.157)
**E. Urban—adjusted for SES** ^3^				
I. Full sample (N = 138,774)				
Child marriage	0.165 ***	Ref.	1.123 ***	1.159 ***
	(0.105, 0.225)		(1.074, 1.174)	(1.091, 1.232)
II. Not pregnant/lactating and had not given birth in the past 5 years (N = 84,805)				
Child marriage	0.157 ***	Ref.	1.088 ***	1.155 ***
	(0.086, 0.229)		(1.030, 1.149)	(1.074, 1.243)
**F. Rural—adjusted for SES**				
I. Full sample (N = 357,509)				
Child marriage	−0.001	Ref.	1.048 ***	1.001
	(−0.030, 0.028)		(1.021, 1.076)	(0.972, 1.031)
II. Not pregnant/lactating and had not given birth in the past 5 years (N = 198,555)				
Child marriage	−0.022	Ref.	1.029 *	0.977
	(−0.059, 0.015)		(0.995, 1.065)	(0.941, 1.014)

^1^ Estimates were obtained using complex survey weights; 95% confidence intervals are in parenthesis; *** *p* < 0.001, * *p* < 0.05. ^2^ The multivariable specifications accounted for age group (18 to 29, 30 to 44, or 45 to 49); religion (Hindu, Muslim, Christian, or other); caste (none, scheduled caste, scheduled tribe, or other backward class); relationship to household head (head, wife, daughter, daughter-in-law, or other); and geographic region fixed effects (north, central, east, northeast, west, or south). ^3^ In the SES specification, in addition to the sociodemographic correlates, the following SES conditions were controlled for: poor and lower education, poor and higher education, non-poor and lower education, and non-poor and higher education.

**Table 7:** Estimates of linear regression and relative risk ratios for facing problems accessing healthcare categorized by score—according to age groups.

	Linear Model	Multinomial Logistic Model		
		Base Outcome:	Outcome I:	Outcome II:
		No/little Problem	Some Problems	Big Problems
**A. Unadjusted** ^1^				
I. Age 18–29 (N = 190,750)				
Child marriage	0.625 ***	Ref.	1.505 ***	1.743 ***
	(0.581, 0.670)		(1.452, 1.561)	(1.674, 1.815)
II. Age 30–44 (N = 152,486)				
Child marriage	0.603 ***	Ref.	1.514 ***	1.673 ***
	(0.563, 0.643)		(1.467, 1.562)	(1.614, 1.734)
II. Age 45–49 (N = 62,968)				
Child marriage	0.315 ***	Ref.	1.275 ***	1.278 ***
	(0.241, 0.388)		(1.203, 1.351)	(1.196, 1.366)
**B. Adjusted** ^2^				
I. Age 18–29 (N = 190,750)				
Child marriage	0.292 ***	Ref.	1.253 ***	1.321 ***
	(0.250, 0.334)		(1.208, 1.300)	(1.267, 1.377)
II. Age 30–44 (N = 152,486)				
Child marriage	0.260 ***	Ref.	1.243 ***	1.271 ***
	(0.221, 0.299)		(1.204, 1.284)	(1.224, 1.320)
II. Age 45–49 (N = 62,968)				
Child marriage	0.040	Ref.	1.075 **	1.008
	(−0.029, 0.110)		(1.014, 1.141)	(0.941, 1.080)
**C. Adjusted for SES** ^3^				
I. Age 18–29 (N = 190,750)				
Child marriage	0.097 ***	Ref.	1.131 ***	1.103 ***
	(0.056, 0.139)		(1.090, 1.175)	(1.057, 1.152)
II. Age 30–44 (N = 152,486)				
Child marriage	0.070 ***	Ref.	1.119 ***	1.081 ***
	(0.032, 0.108)		(1.080, 1.160)	(1.037, 1.127)
II. Age 45–49 (N = 62,968)				
Child marriage	−0.098 ***	Ref.	0.970	0.884 ***
	(−0.165, −0.030)		(0.913, 1.030)	(0.825, 0.948)

^1^ Estimates were obtained using complex survey weights; 95% confidence intervals are in parenthesis; *** *p* < 0.001, ** *p* < 0.01 ^2^ The multivariable specifications accounted for residence (urban or rural); religion (Hindu, Muslim, Christian, or other); caste (none, scheduled caste, scheduled tribe, or other backward class); relationship to household head (head, wife, daughter, daughter-in-law, or other); and geographic region fixed effects (north, central, east, northeast, west, or south). ^3^ In the SES specification, in addition to the sociodemographic correlates, the following SES conditions were controlled for: poor and lower education, poor and higher education, non-poor and lower education, and non-poor and higher education.

**Table 8:** Estimates of the hurdle model.

	Part 1:	Part 2:
	Probability of Facing Big Problems	Number of Big Problems
**A. Unadjusted** ^1^		
I. Full sample (N = 496,283)		
Child marriage	1.389 ***	1.084 ***
	(1.359, 1.419)	(1.075, 1.093)
II. Not pregnant/lactating and had not given birth in the past 5 years (N = 283,360)		
Child marriage	1.367 ***	1.080 ***
	(1.331, 1.405)	(1.068, 1.092)
**B. Adjusted** ^2^		
I. Full sample (N = 496,283)		
Child marriage	1.155 ***	1.039 ***
	(1.130, 1.180)	(1.031, 1.048)
II. Not pregnant/lactating and had not given birth in the past 5 years (N = 283,360)		
Child marriage	1.147 ***	1.038 ***
	(1.116, 1.179)	(1.026, 1.049)
**C. Adjusted for SES** ^3^		
I. Full sample (N = 496,283)		
Child marriage	1.049 ***	1.007 *
	(1.026, 1.072)	(0.999, 1.015)
II. Not pregnant/lactating and had not given birth in the past 5 years (N = 283,360)		
Child marriage	1.044 ***	1.008
	(1.015, 1.073)	(0.998, 1.019)

^1^ Estimates were obtained using complex survey weights; 95% confidence intervals are in parenthesis; *** *p* < 0.001, * *p* < 0.05. ^2^ The multivariable specifications accounted for age group (18 to 29, 30 to 44, or 45 to 49); residence (urban or rural); religion (Hindu, Muslim, Christian, or other); caste (none, scheduled caste, scheduled tribe, or other backward class); relationship to household head (head, wife, daughter, daughter-in-law, or other); and geographic region fixed effects (north, central, east, northeast, west, or south). ^3^ In the SES specification, in addition to the sociodemographic correlates, the following SES conditions were controlled for: poor and lower education, poor and higher education, non-poor and lower education, and non-poor and higher education.

## Data Availability

The dataset used in this study is available from the USAID’s Demographic and Health Surveys (DHS) program. The DHS datasets are free to download and use upon registering via the DHS program website: https://dhsprogram.com/data/new-user-registration.cfm. Accessed on 6 January 2022.
